# Maximizing sprint performance among adolescent sprinters: a controlled evaluation of functional, traditional, and combined training approaches

**DOI:** 10.3389/fpubh.2025.1596381

**Published:** 2025-05-12

**Authors:** Xiaohuan Liu, Yongjing Shao, Sohom Saha, Zijian Zhao, Debajit Karmakar

**Affiliations:** ^1^School of Physical Education, Shaoguan University, Shaoguan, China; ^2^Sport and Fitness Sciences, Gdansk University of Physical Education and Sport, Gdańsk, Poland; ^3^Department of Sport Psychology, Lakshmibai National Institute of Physical Education, Gwalior, Madhya Pradesh, India; ^4^Department of Physical Education, Zhengzhou University, Zhengzhou, India; ^5^Department of Physical Education Pedagogy, Lakshmibai National Institute of Physical Education, Gwalior, Madhya Pradesh, India

**Keywords:** functional training, traditional training, combined training, sprinting performance, physical fitness, skill-related performance

## Abstract

**Background:**

Sprint performance plays a crucial role in competitive sports, particularly among adolescent athletes. Training methodologies such as Functional Training (FT), Traditional Training (TT), and a Combined Training (CT) approach have been widely implemented to improve sprinting ability, agility, and coordination. However, the comparative effectiveness of these methods remains inconclusive. This study aims to evaluate the differential impact of FT, TT, and CT on key physiological and skill-related performance variables in adolescent sprinters.

**Methods:**

A total of 52 national-level adolescent sprinters (aged 15–18 years) were randomly assigned to four groups: Functional Training Group (FTG, *n* = 13), Traditional Training Group (TTG, *n* = 13), Combined Training Group (CTG, *n* = 13), and Control Group (CG, *n* = 13). The intervention lasted for 8 weeks, with training sessions conducted 6 days a week. Sprint performance, agility, coordination, VO₂ max, muscular strength, and body fat percentage were assessed pre- and post-intervention. A repeated-measures ANOVA was used to analyze within- and between-group differences, with significance set at *p* < 0.05.

**Results:**

Combined Training Group demonstrated the most significant improvements across all performance variables, including VO₂ max (+4.32%), muscular strength (+8.93%), and sprinting ability (−4.71%). FTG showed substantial gains in agility (−2.16%) and coordination (+4.40%), whereas TTG exhibited moderate improvements in strength (+1.43%) and sprint time (−2.18%). The CG group showed no significant changes. Statistical analysis confirmed a significant main effect of training interventions (*F* = 72.34, *p* < 0.001, *η*^2^_p_ = 0.81), highlighting the superior efficacy of CTG.

**Discussion:**

The findings suggest that a combined approach integrating FT and TT yields optimal improvements in sprint performance by enhancing both neuromuscular coordination and force production. While FT alone is effective in refining agility and coordination, TT primarily contributes to strength gains. The absence of structured training in the CG reinforces the necessity of targeted interventions for performance enhancement. These results provide valuable insights for coaches and sports scientists in designing sprint training programs for adolescent athletes. Future studies could explore the long-term effects of combined FT and TT interventions across different age groups and genders to assess their generalizability.

## 1 Introduction

In the realm of elite athletics, sprinting stands out as a discipline where fractions of a second can demarcate triumph from defeat. Recent analyses of World Championship and Olympic performances (2016–2023) indicate that the margin of victory in 100 m finals has averaged just 0.07 s, underscoring the critical importance of adopting the most effective training methodologies (1). Athletic organizations maintain performance optimization as their driving force to use innovative training methods as sports scientists and coaches continuously improve sprinter abilities (2). Among these strategies, functional training and traditional training have gained increasing prominence in recent years (3, 4). Functional training, rooted in the principle of specificity, aims to replicate the biomechanical and neuromuscular demands of sprinting through exercises that closely mimic the sport’s movement patterns (5). Functional training bases its approach on the Specific Adaptation to Imposed Demands (SAID) principle by focusing on exactly how the neuromuscular system adapts. Research utilizing EMG studies demonstrates that the nervous system activates motor units through precise coordination for sprint-specific actions which result in 95% activation of fast-twitch fibers (6). Functional training produces significant changes in motor unit synchronization that amount to 23–28% compared to initial measurements in muscles such as gastrocnemius and vastus lateralis (7). Traditional training chooses systematic overload methods that use isolated machine-based or free-weight exercises to achieve general strength development through linear movement patterns. Muscular development and structural adaptations make up the primary focus of this approach while the flexibility of fascicle length and pennation angle in sprint relevant muscles increases by 12–15% through this methodology (8). Medical research reveals that force production enhances by 18–22% during the acceleration phases of sprinting (9). The comparison requires attention because different training approaches base their fundamental concepts on opposing principles while focusing on separate neuromuscular developmental procedures.

The physiological mechanisms underlying both approaches differ significantly in their impact on neuromuscular adaptation. Functional training primarily enhances neural drive efficiency through movement-specific patterns, with studies showing a 31% improvement in rate of force development (RFD) during sprint-specific movements (10). This adaptation is attributed to enhance motor unit synchronization and reduced neural inhibition during complex, multi-joint movements. Traditional training, meanwhile, induces greater morphological adaptations, with documented increases of 8–12% in cross-sectional area of prime mover muscles and corresponding improvements in absolute strength measures (11). The debate surrounding the relative efficacy of these training methods has intensified as new research emerges on the specific adaptations they induce. Recent longitudinal studies tracking elite sprinters over multiple seasons (2018–2023) have shown that programs emphasizing functional training reported a 2.8% greater improvement in 0–30 m acceleration times compared to traditional training-focused programs (12). However, traditional training programs demonstrated superior results in maximum strength parameters, with athletes showing 15–20% greater improvements in squat and deadlift performance (13). The scientific rationale for investigating both methodologies is further strengthened by recent advances in our understanding of sprint-specific neural adaptations. Functional training has been shown to enhance motor unit recruitment patterns specific to sprinting, with high-speed electromyography revealing a 24% improvement in muscle activation synchronization during the critical first 10 meters of acceleration (14). Traditional training, while producing different neural adaptation patterns, has demonstrated significant benefits in developing the fundamental force-producing capabilities necessary for sprint performance, with studies showing improvements of 16–20% in ground reaction forces during the acceleration phase (15). On the other hand, traditional training methods have a long-standing history in strength and conditioning for sprinters. This approach typically involves more isolated exercises, often using weight machines or free weights, and focuses on developing overall strength and power in key muscle groups (4). The rationale behind traditional training is that by increasing an athlete’s general strength and power capacity, they will be better equipped to generate the forces necessary for explosive sprint performance (16). The inclusion of both methods in this study is justified by the conflicting evidence in the current literature. While some studies have shown superior results with functional training for improving sport-specific performance (17), others have demonstrated significant benefits from traditional strength training for sprint speed and acceleration (18). By directly comparing these methods within the same study, using a cohort of trained sprinters, we aim to provide clarity on their relative effectiveness. Researchers establish the need for more exploration about functional training and traditional training because their separate and combined contributions to sprint performance remain uncertain despite recent empirical study interest.

Numerous studies have explored how functional and traditional training impact different facets of athletic performance, with particular attention to sprinting abilities. However, the findings have been mixed, and significant gaps in our understanding persist. Loturco et al. (14) compared the effects of traditional strength training and functional power training on sprint performance in elite young soccer players. Their results indicated that both methods led to improvements in sprint times, but functional power training resulted in greater enhancements in acceleration capabilities. However, this study was limited to soccer players and did not specifically focus on trained sprinters. Wang et al. (19) conducted a study on “Effects of Functional Strength Training Combined with Aerobic Training on Body Composition, Physical Fitness, and Movement Quality in Obese Adolescents. McBride et al. (20) investigated the effects of traditional resistance training on sprint performance and found significant improvements in both acceleration and maximum velocity phases. Yet, their study did not include a comparison with functional training methods, leaving questions about the relative efficacy of the two approaches unanswered. A significant gap identified across multiple studies is the limited focus on skill-related performance metrics beyond basic sprint times. While improvements in strength and power are frequently reported, there is a scarcity of research examining how different training methodologies affect crucial technical aspects of sprinting such as stride length, stride frequency, and ground contact times (21, 22). Another notable gap lies in the long-term effects of these training methods. Most studies have been relatively short-term (8–12 weeks), providing limited insight into how the adaptations to functional and traditional training may differ over extended periods (23). This is particularly relevant for sprinters, whose careers often span many years and require carefully periodized training programs. Furthermore, there is a lack of research examining the potential synergistic effects of combining functional and traditional training methods in sprinters’ programs. While some studies have explored hybrid approaches in other sports, the optimal integration of these methodologies for sprint performance remains unclear (24). This research aims to bridge critical gaps in understanding how distinct training methodologies influence both the physical and technical aspects of sprint performance in adolescent athletes. By assessing a comprehensive set of performance indicators including sprint times, acceleration profiles, peak velocity, and biomechanical factors such as stride mechanics and ground contact times this study provides evidence-based insights into the most effective training approaches for this developmental stage. The findings contribute to the growing body of knowledge on youth sprint training adaptations and offer practical guidance for coaches and practitioners seeking to optimize performance outcomes in adolescent sprinters.

## 2 Materials and methods

### 2.1 Experimental design

**Figure 1:**
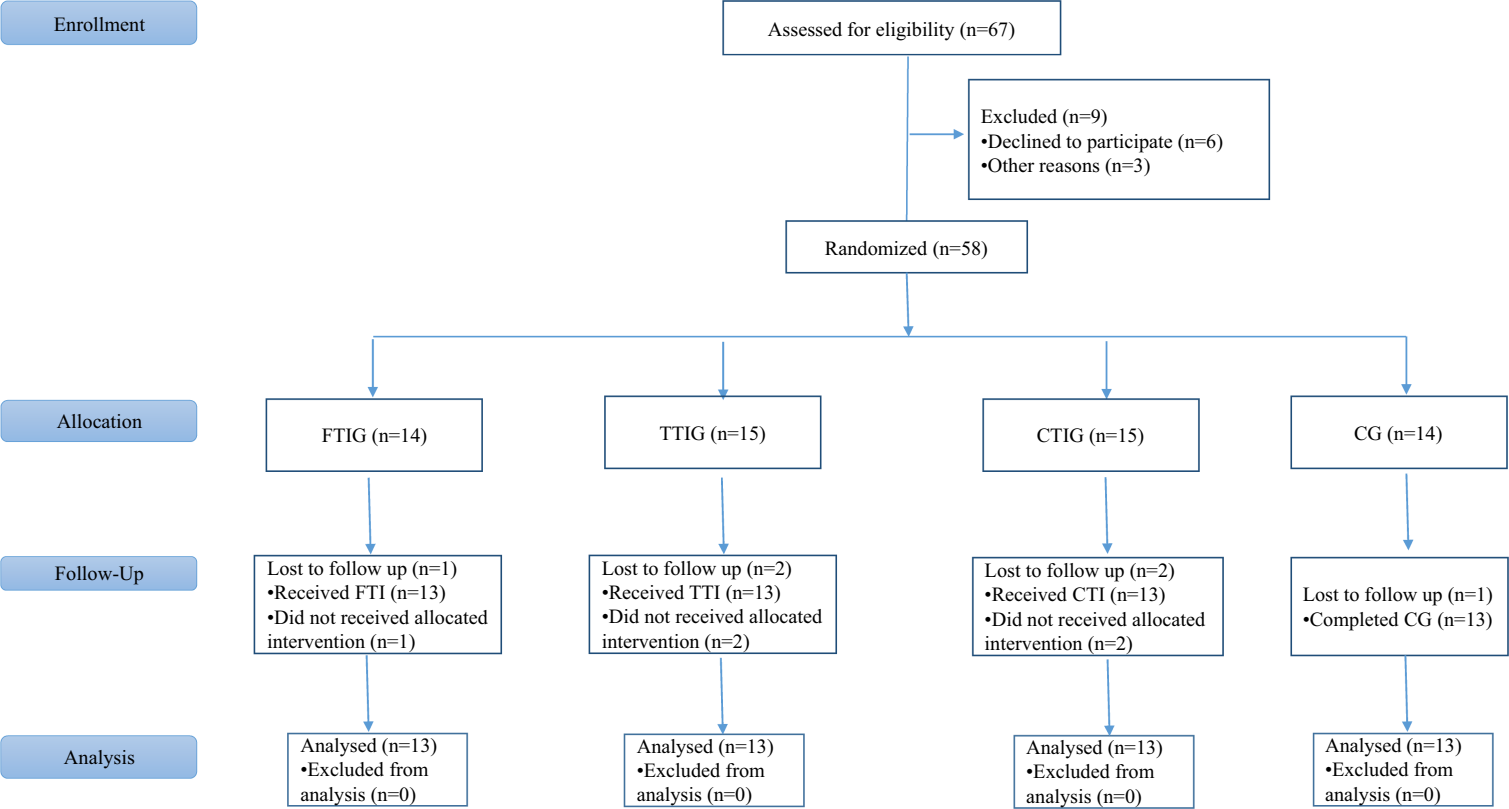
Flowchart of the trial from the baseline.

This study follows a randomized controlled trial (RCT) design involving 52 National Level adolescent sprinters belongs to Zhumadian No. 8 Junior High School, Zhumadian City, Henan Province, China. Age range of the participants were 15–18 years. Participants were randomly assigned to one of four groups: Functional Training Group (FTG) (Table 1), Traditional Training Group (TTG) (Table 2), Combined Training Group (CTG) (Table 3) and Control Group (CG), without any statistical differences within them, as shown in Table 4. The FTG will undergo a structured 8-week functional training program, while the TTG will follow a traditional strength and conditioning program over the same period, as shown in Figure 1. The CTG will participate in a periodized program combining both functional and traditional training elements, but control group did not take part in any training activities. All training groups engaged in training 6 days a week from 23/05/2024 to 18/07/2024. All training sessions for the experimental groups will be supervised by certified strength and conditioning specialists to ensure protocol adherence. The interventions for both experimental groups will be matched in terms of training volume and intensity to eliminate potential confounding effects related to the training load.

**Table 1:** Functional training plan.

Week	Focus area	Day 1	Day 2	Day 3	Day 4	Day 5	Day 6 and 7
1	Adaptation and Mobility	**Core Stability and Mobility** Planks (3 × 30s), Leg Raises (3×20), Glute Bridges (3 × 20)	**Lower Body Strength and Mobility** Squats (3 × 12), Lunges (3 × 10 each leg), Mobility drills	**Sprint Technique** High Knee Drills (5 × 20 m), Acceleration Drills (5 × 20 m), Core (V-Ups 3 × 15)	**Upper Body Strength** Push-ups (3 × 15), Pull-ups (3 × 8), Medicine Ball Throws (3 × 10)	**Recovery Run and Stretching** Light jog (20 min), Stretching (3 × 30s per muscle)	Rest
2	Mobility and Strength Endurance	**Plyometrics and Core** Box Jumps (3 × 10), Depth Jumps (3 × 8), Planks (3 × 40s)	**Lower Body Strength and Resisted Sprints** Weighted Squats (3 × 12), Resisted Sprints (5 × 30 m)	**Sprint Technique** Acceleration Drills (5 × 30 m), Strides (4 × 40 m), Core (Russian Twists 3 × 20)	**Upper Body Strength** Push Press (3 × 10), Push-ups (4 × 15)	**Recovery Run and Stretching** Light jog (15 min), Stretching (3 × 30s per muscle)	Rest
3	Core Stability and Agility	**Speed Technique and Core** Acceleration Drills (5 × 20 m), Core (V-Ups 3 × 20), Planks (3 × 45s)	**Lower Body Strength** Split Squats (3 × 12), Mobility drills (Lunges with Rotation)	**Sprint Technique** 6 × 40 m sprints with 2 min rest, Core (Leg Raises 3 × 25)	**Upper Body Strength and Plyometrics** Push-ups (4 × 15), Pull-ups (4 × 10), Bounding (3 × 8)	**Active Recovery and Flexibility** Swimming (20 min), Flexibility (3 × 30s per muscle)	Rest
4	Power Development and Speed	**Plyometrics and Sprint Technique** Box Jumps (4 × 10), Depth Jumps (4 × 8), Acceleration (5 × 50 m)	**Lower Body Power** Squat Jumps (4 × 8), Sprint Drills (5 × 60 m with 2 min rest)	**Sprint Technique** 6 × 50 m with 2 min rest, Core (Planks 4 × 1 min)	**Upper Body Power** Push Press (4 × 12), Medicine Ball Throws (4 × 12)	**Recovery Run and Stretching** Light jog (20 min), Stretching (3 × 30s per muscle)	Rest
5	Speed-Endurance and Agility	**Speed Technique and Core** Max Velocity Drills (5 × 40 m), Core (Russian Twists 4 × 20), Planks (4 × 1 min)	**Lower Body Strength and Agility** Squats (4 × 12), Agility drills (Cone drills 4 × 5)	**Sprint Technique** 6 × 60 m with 2.5 min rest, Core (Leg Raises 4 × 25)	**Upper Body Strength and Plyometrics** Push-ups (4 × 20), Pull-ups (4 × 12), Bounding (4 × 10)	**Light Recovery Run and Stretching** Jog (20 min), Flexibility (3 × 30s per muscle)	Rest
6	Strength and Power	**Plyometrics and Core** Box Jumps (4 × 12), Planks (4 × 1 min), Acceleration Drills (5 × 50 m)	**Lower Body Power and Sprint Drills** Squat Jumps (4 × 10), Sprint Drills (5 × 80 m with 2.5 min rest)	**Sprint Technique and Core** 5 × 70 m sprints, Core (V-Ups 4 × 20)	**Upper Body Power** Push Press (4 × 12), Medicine Ball Throws (4 × 12)	**Recovery Run and Stretching** Jog (20 min), Flexibility (3 × 30s per muscle)	Rest
7	Speed-Endurance and Recovery	**Speed Technique and Core** Max Velocity Drills (5 × 50 m), Planks (4 × 45s), Leg Raises (4 × 25)	**Lower Body Strength and Agility** Squats (4 × 15), Agility drills (Ladder drills 4 × 5)	**Sprint Technique and Core** 6 × 70 m with 2.5 min rest, Core (V-Ups 4 × 25)	**Upper Body Strength** Push-ups (4 × 20), Pull-ups (4 × 15)	**Light Recovery Run and Flexibility** Jog (20 min), Flexibility (3 × 30s per muscle)	Rest
8	Power and Max Speed Development	**Plyometrics and Acceleration** Box Jumps (4 × 15), Depth Jumps (4 × 10), Acceleration Drills (5 × 70 m)	**Lower Body Power and Sprint Drills** Squat Jumps (4 × 12), Sprint Drills (5 × 100 m with 3 min rest)	**Sprint Technique and Core** 5 × 80 m sprints, Core (Leg Raises 4 × 25)	**Upper Body Power** Push Press (4 × 15), Medicine Ball Throws (4 × 15)	**Recovery Run and Stretching** Jog (20 min), Flexibility (3 × per muscle)	Rest

**Table 2:** Traditional training plan.

Week	Training focus	Days/week	Session breakdown	Volume	Intensity
1	General Conditioning	5	- Warm-up (dynamic stretches, mobility drills) - Aerobic base building (steady-state running, 20–30 min) - Core stability exercises - Cool-down (static stretches)	60–75 min/session	Low to moderate
2	Strength Endurance	5	- Warm-up - Hill sprints (50 m × 6–8) - Resistance training (bodyweight circuits) - Core strength drills - Cool-down	60–75 min/session	Moderate
3	Strength and Power	5	- Warm-up - Plyometric training (bounding, box jumps) - Resistance training (moderate weight, high reps) - Core stability drills - Cool-down	60–75 min/session	Moderate to High
4	Speed Development	5	- Warm-up - Acceleration drills (10–30 m sprints) - Resisted sprints (sled pulls) - Sprint mechanics drills - Cool-down	75–90 min/session	High
5	Strength and Power	5	- Warm-up - Olympic lifts (cleans, snatches) - Plyometric training (depth jumps) - Resistance training (high weight, low reps) - Cool-down	75–90 min/session	High
6	Speed Endurance	5	- Warm-up - Speed endurance drills (150 m–300 m intervals) - Resistance training (bodyweight + low resistance circuits) - Core work - Cool-down	75–90 min/session	High
7	Technical Skills	5	- Warm-up - Sprint start practice (blocks) - Maximal velocity runs (40 m–60 m) - Sprint technique drills - Cool-down	75–90 min/session	High
8	Speed and Strength Maintenance	5	- Warm-up - Speed drills (10 m–40 m acceleration sprints) - Resistance training (low volume, moderate weight) - Core stabilization drills - Cool-down	60–75 min/session	Moderate

**Table 3:** Combined training group.

Week	Focus area	Day 1	Day 2	Day 3	Day 4	Day 5	Day 6 and 7
1	Adaptation and Conditioning	Core Stability and Mobility; Planks (3 × 30s), Leg Raises (3 × 20)	Aerobic Base Building; Steady-State Run (30 min)	Sprint Technique; High Knees (5 × 20 m)	Upper Body Strength; Push-ups (3 × 15), Pull-ups (3 × 8)	Light Recovery Run and Stretching (20 min)	Rest
2	Strength Endurance	Plyometrics and Core; Box Jumps (3 × 10), Planks (3 × 40s)	Hill Sprints (50 m × 6), Resistance Circuits	Sprint Technique; Acceleration Drills (5 × 30 m)	Upper Body Strength; Push Press (3 × 10), Push-ups (4 × 15)	Recovery Run and Stretching (15 min)	Rest
3	Core Stability and Agility	Speed Technique; Acceleration Drills (5 × 20 m), Core	Lower Body Strength; Split Squats (3 × 12)	Sprint Technique; 6 × 40 m Sprints	Upper Body Strength; Push-ups (4 × 15), Bounding (3 × 8)	Swimming Recovery (20 min)	Rest
4	Power Development	Plyometrics; Box Jumps (4 × 10), Depth Jumps	Lower Body Power; Squat Jumps (4 × 8)	Sprint Technique; 6 × 50 m Sprints	Upper Body Power; Push Press (4 × 12)	Recovery Run and Stretching (20 min)	Rest
5	Speed-Endurance and Agility	Max Velocity Drills; Core (Russian Twists 4 × 20)	Lower Body Strength; Squats (4 × 12)	Sprint Technique; 6 × 60 m Sprints	Upper Body Strength and Plyo; Push-ups (4 × 20), Bounding (4×10)	Light Recovery Run and Flexibility	Rest
6	Strength and Power	Plyometrics; Box Jumps (4 × 12), Acceleration Drills	Lower Body Power; Sprint Drills (5 × 80 m)	Sprint Technique; 5 × 70 m Sprints	Upper Body Power; Push Press (4 × 12)	Recovery Run and Stretching (20 min)	Rest
7	Speed-Endurance and Recovery	Max Velocity Drills; Core (Planks 4 × 45s)	Lower Body Strength and Agility; Squats (4 × 15)	Sprint Technique; 6 × 70 m Sprints	Upper Body Strength; Push-ups (4 × 20), Pull-ups (4 × 15)	Light Recovery Run and Flexibility	Rest
8	Max Speed and Power	Plyometrics and Acceleration; Box Jumps (4 × 15)	Lower Body Power; Sprint Drills (5 × 100 m)	Sprint Technique; 5 × 80 m Sprints	Upper Body Power; Medicine Ball Throws (4 × 15)	Recovery Run and Stretching (20 min)	Rest

**Table 4:** Demographic values for participants.

Measures	FTG Mean ± SD	TTG Mean ± SD	CTG Mean ± SD	CG Mean ± SD
Age (Yrs)	16.69 ± 1.03	16.38 ± 1.19	16.76 ± 1.30	16.38 ± 1.12
Weight (kg)	60.41 ± 1.74	60.01.53 ± 2.31	61.52 ± 2.53	60.05 ± 2.77
Height (cm)	156.76 ± 2.61	157 ± 2.76	157.07 ± 2.92	157 ± 2.79
Experience (years)	4.07 ± 0.75	4.23 ± 0.83	3.61 ± 0.76	3.92 ± 0.75

### 2.2 Outcome assessment

Primary outcomes are divided into two categories: Physical Fitness Variables and Skill Related Performance Variables.

#### 2.2.1 Physical fitness variables

VO2 Max: measured using a graded exercise test on a treadmill with gas analysis via the COSMED K5 portable metabolic system (25), which has established validity and reliability. Participants were instructed to run to volitional fatigue following a ramp protocol, with the highest oxygen uptake recorded as VO₂ max (mL/kg/min).

Lower body strength: maximal lower-body strength was assessed using a direct 1-repetition maximum (1RM) leg press test (26), following the standardized protocol outlined by Baechle and Earle (26). Each participant completed a warm-up consisting of two sets of 8–10 repetitions at approximately 40–60% of their perceived maximum load. Subsequently, the load was progressively increased over a maximum of five attempts to identify the heaviest weight that could be lifted once with proper technique. A rest interval of 3 min was provided between each attempt to ensure adequate recovery and test accuracy. Importantly, no predictive equations were used; the 1RM value was determined through direct measurement to ensure maximal validity.

Body Fat Percentage: Measured via dual-energy X-ray absorptiometry (DEXA) (27) scanning, which provides high-precision body composition analysis. Participants were instructed to fast for at least 3 h and avoid exercise 12 h before testing to ensure consistency.

#### 2.2.2 Skill-related performance variables

Sprinting ability: sprinting performance was assessed using a 30-meter sprint test. Electronic timing gates were positioned at the 0-meter and 30-meter marks to ensure precise and reliable time measurement (28). Each athlete performed three maximal-effort trials, with two-minute rest intervals between each attempt. The fastest time recorded across the trials was used for further analysis.

Agility: agility was evaluated using the Illinois Agility Test, conducted in accordance with the standard testing protocol (29). To ensure accurate measurement, electronic timing gates were placed at the starting and finishing lines. Each participant completed two trials, separated by a three-minute rest interval. The better of the two recorded times was used for statistical analysis.

Coordination: coordination was measured using a custom-designed foot-eye coordination task specifically developed for adolescent athletes. The task involved following a visually guided LED sequence on a footwork panel, emphasizing both accuracy and response time (30). The total coordination score was computed as a composite of the number of correct responses and time taken. Each participant performed two trials with a two-minute rest period between them, and the higher score was retained for final analysis.

Assessments will be performed at baseline (Pre-intervention) and after the intervention (8 weeks). Trained assessors, unaware of the participants’ group assignments, will conduct all measurements to reduce potential bias.

### 2.3 Ethical approval and consent

This study received approval from the Institutional Ethical Committee of Zhumadian No. 8 Junior High School (Approval No. IEC/ZJS/SPE/475). It adheres to the ethical guidelines specified in the Declaration of Helsinki, emphasizing respect for human participants, confidentiality, voluntary involvement, and the right to withdraw at any point without repercussion (31). Prior to the commencement of the study, both adolescent athletes and their guardians were thoroughly informed about the study’s objectives, procedures, potential risks, and benefits. A comprehensive information sheet detailing the nature of the interventions and assessments was provided to ensure transparency. To secure informed consent, athletes and their guardians were encouraged to ask questions and seek clarification from the research team. Written informed consent was obtained from both the athletes and their legal guardians, who voluntarily signed consent forms after being assured of their right to withdraw from the study at any time without any impact on their training programs or athletic development. All personal data and performance outcomes were kept strictly confidential, with participants identified through unique codes for data collection and analysis.

### 2.4 Statistical analysis

The statistical analysis was conducted to evaluate the effects of functional training (FTG), traditional training (TTG), and combined training (CTG) compared to a control group (CG) on selected physiological parameters. All variables were normally distributed (Shapiro–Wilk test). Descriptive statistics, including mean and standard deviation (Mean ± S.D.), were calculated for pre and post intervention measurements across all groups. A repeated-measures analysis of variance (ANOVA) was used to determine the within-group and between-group differences. The significance level was set at *p* < 0.05, with partial eta squared (*η*^2^_p_) calculated to assess effect sizes. *η*^2^_p_ of measures was counted as small: less than 0.06, moderate: between 0.06 to 0.13 and large: that was 0.14 or more (32).

## 3 Results

**Figure 2:**
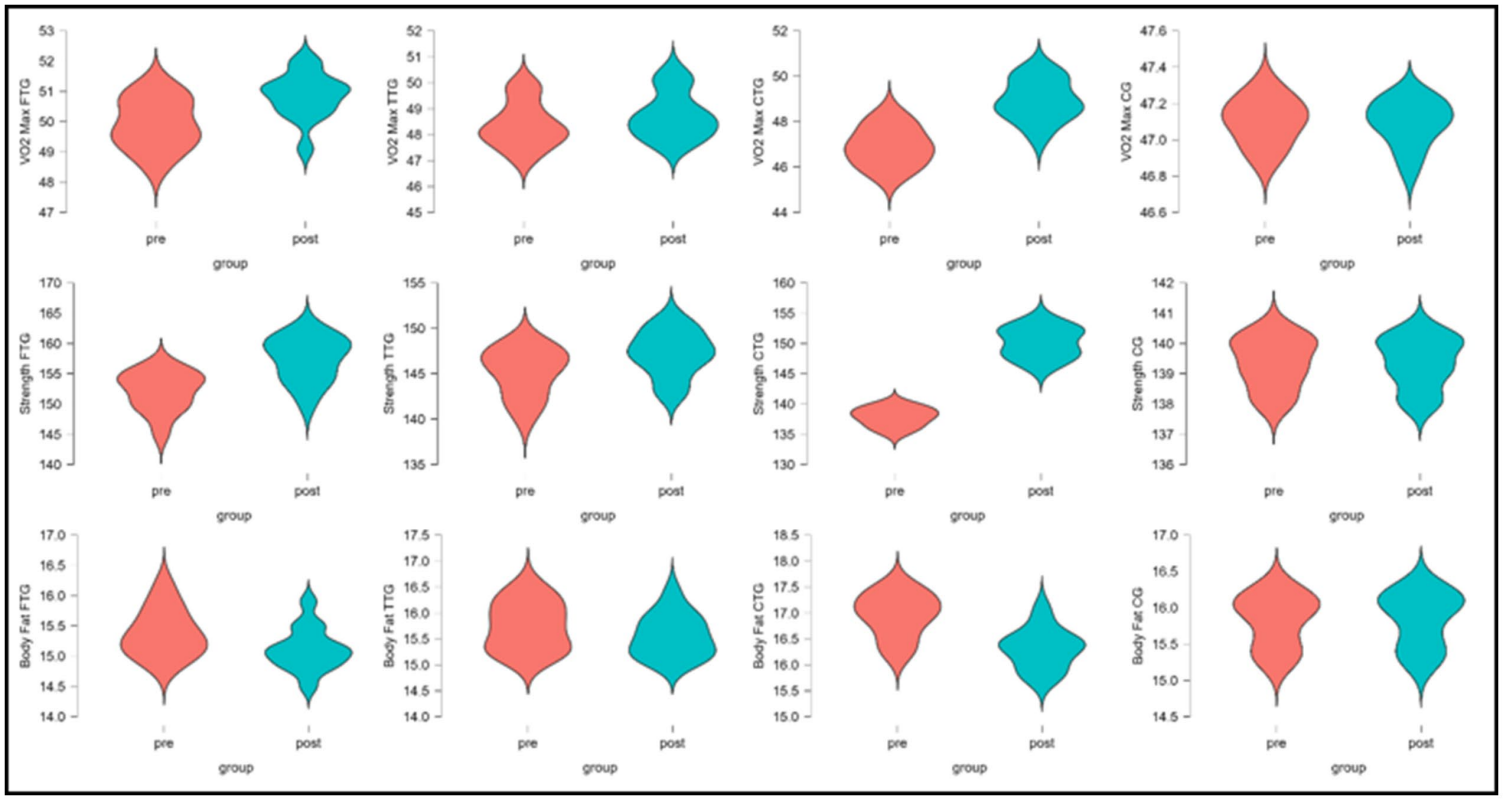
Pre- and post-intervention distributions of physiological parameters across training groups (FTG, TTG, CTG, CG).

**Figure 3:**
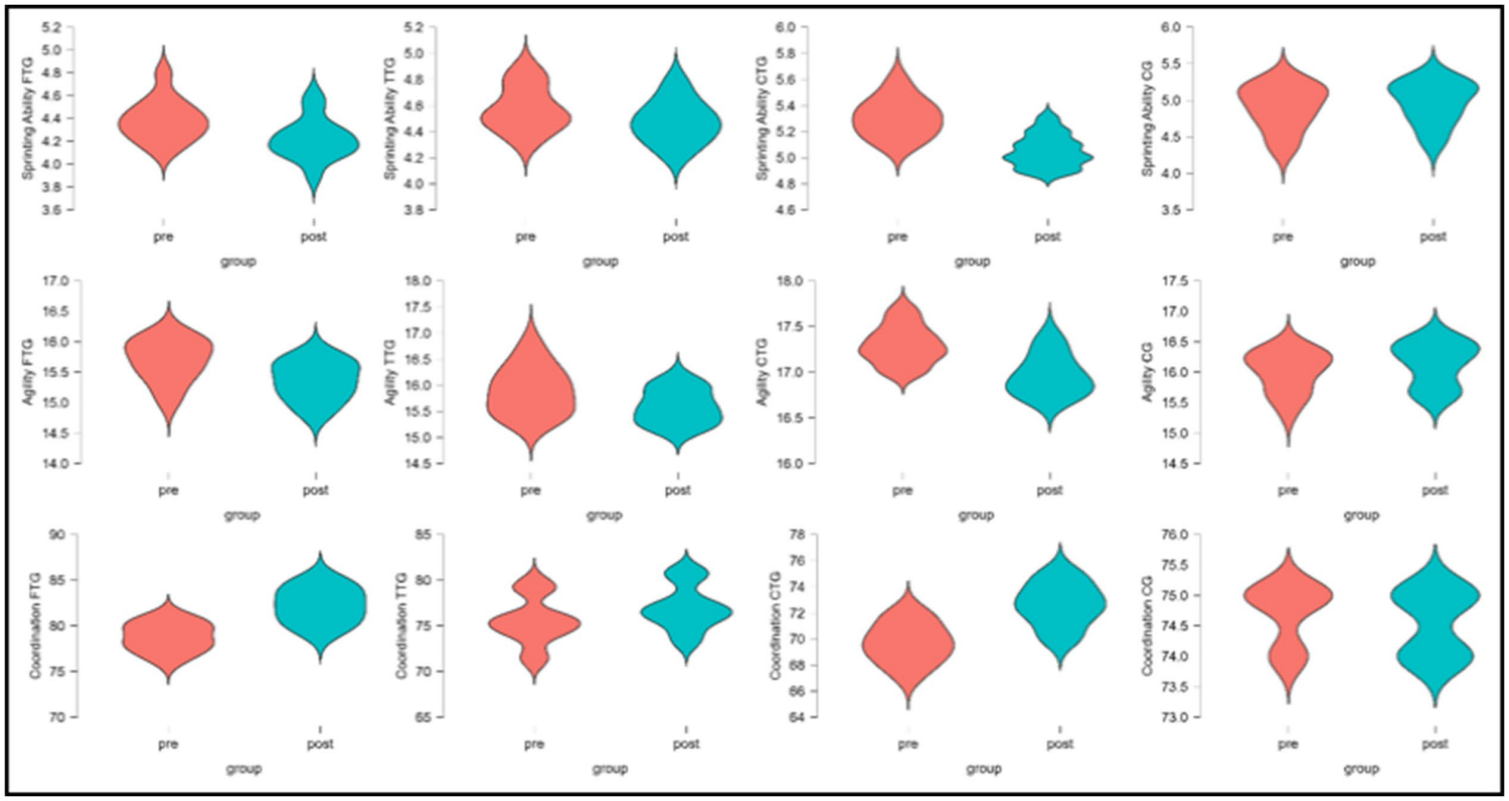
Pre- and Post-Intervention Distributions of Skill-Related Performance Parameters across Training Groups (FTG, TTG, CTG, CG).

The data presented in Table 5 evaluates the impact of functional training (FTG), traditional training (TTG), combined training (CTG), and a control group (CG) on various physiological and performance-related variables: VO₂ max, strength, body fat percentage, sprinting ability, agility, and coordination. Each variable was assessed using mean and standard deviation (Mean ± S.D.) values for pre- and post-intervention measurements. Statistical parameters such as sum of squares (SS), *F*-values, *p*-values, and partial eta squared (*η*^2^_p_) were computed to determine the significance and effect sizes of the interventions. The results demonstrated significant differences across all measured variables (*p* < 0.001) with substantial effect sizes. For cardiorespiratory fitness (VO2 Max), a significant main effect was observed (*F* = 54.44, p < 0.001, *η*^2^_p_ = 0.77), with the CTG showing the greatest improvement (4.32%), followed by FTG (1.84%), and TTG (0.76%), while the CG showed a marginal decline (−0.02%). Muscular strength analyses revealed a robust main effect (*F* = 140.87, *p* < 0.001, *η*^2^_p_ = 0.89), with the CTG demonstrating the most substantial strength gains (8.93%), followed by FTG (3.49%) and TTG (1.43%), while the CG maintained baseline levels (0.00%). Body composition improvements were significant (*F* = 49.402, *p* < 0.001, *η*^2^_p_ = 0.75), with the CTG achieving the greatest reduction in body fat percentage (−3.83%), followed by FTG (−1.75%) and TTG (−1.20%), while the CG showed a slight increase (0.12%). Sprint performance exhibited significant improvements (*F* = 72.34, *p* < 0.001, *η*^2^_p_ = 0.81), with the CTG showing the largest reduction in sprint time (−4.71%), followed by FTG (−3.65%) and TTG (−2.18%), while the CG demonstrated performance deterioration (1.22% increase in time). Agility measurements revealed significant changes (*F* = 18.65, *p* < 0.001, *η*^2^_p_ = 0.53), with FTG showing the greatest improvement (−2.16%), followed closely by CTG (−1.96%), while TTG showed minimal change (0.12%), and CG demonstrated slight performance decline (0.81% increase). Coordination capacity showed significant enhancement (*F* = 96.14, *p* < 0.001, *η*^2^_p_ = 0.85), with FTG demonstrating the greatest improvement (4.40%), followed closely by CTG (4.18%) and TTG (1.94%), while CG showed a slight decline (−0.21%) (Figures 2, 3).

**Table 5:** Impact of functional, traditional, and combined training on physiological and performance variables (mean ± S.D.) with statistical analysis.

Variables	Groups	Pre data (Mean ± S.D.)	Post data (Mean ± S.D.)	Δ (%)	SS	*F*	*p*	*η* ^2^_p_
VO2 Max	FTG	49.93 ± 0.83	50.85 ± 0.76	1.84%	15.39	54.44	<0.001	0.77
	TTG	48.37 ± 0.9	48.74 ± 0.91	0.76%				
	CTG	46.9 ± 0.86	48.93 ± 0.9	4.32%				
	CG	47.1 ± 0.13	47.09 ± 0.12	−0.02%				
Strength	FTG	152.07 ± 3.3	157.38 ± 3.66	3.49%	565.61	140.87	<0.001	0.89
	TTG	145.15 ± 2.64	147.23 ± 2.52	1.43%				
	CTG	137.84 ± 1.57	150.15 ± 2.51	8.93%				
	CG	139.35 ± 0.83	139.35 ± 0.84	0.00%				
Body Fat	FTG	15.38 ± 0.4	15.11 ± 0.36	−1.75%	1.5	49.402	<0.001	0.75
	TTG	15.74 ± 0.46	15.55 ± 0.41	−1.20%				
	CTG	16.95 ± 0.42	16.3 ± 0.39	−3.83%				
	CG	15.81 ± 0.36	15.83 ± 0.37	0.12%				
Sprinting Ability	FTG	4.38 ± 0.18	4.22 ± 0.18	−3.65%	0.35	72.34	<0.001	0.81
	TTG	4.57 ± 0.17	4.47 ± 0.16	−2.18%				
	CTG	5.3 ± 0.15	5.05 ± 0.12	−4.71%				
	CG	4.9 ± 0.3	4.96 ± 0.28	1.22%				
Agility	FTG	15.69 ± 0.34	15.35 ± 0.32	−2.16%	1.01	18.65	<0.001	0.53
	TTG	15.9 ± 0.46	15.92 ± 0.32	0.12%				
	CTG	17.3 ± 0.21	16.96 ± 0.22	−1.96%				
	CG	16 ± 0.34	16.13 ± 0.35	0.81%				
Coordination	FTG	78.76 ± 1.58	82.23 ± 1.96	4.40%	51.3	96.14	<0.001	0.85
	TTG	75.53 ± 2.63	77 ± 2.48	1.94%				
	CTG	69.69 ± 1.54	72.61 ± 1.66	4.18%				
	CG	74.69 ± 0.48	74.53 ± 53	−0.21%				

## 4 Discussion

The primary objective of this study was to investigate the comparative effectiveness of Functional Training (FT), Traditional Training (TT), and Combined Training (CT) on enhancing both physical fitness and skill-related performance in adolescent sprinters. The findings revealed that the CT group demonstrated the most significant improvements across all measured variables, including VO₂ max, muscular strength, body fat percentage, sprinting ability, agility, and coordination. FT showed notable gains in agility and coordination, while TT led to moderate improvements in strength and sprint performance. The control group exhibited no significant changes. The observed differences in VO2 max improvements among the groups is attributed to the distinct physiological adaptations elicited by the varying training modalities. The combined traditional and functional training group demonstrated the most significant increase in VO2 max, likely due to the synergistic effects of the two approaches (33). Traditional strength training primarily targets the development of muscle strength and power through hypertrophy and neuromuscular adaptations, while functional training emphasizes dynamic, multi-joint movements that enhance neuromuscular coordination, proprioception, and cardiovascular engagement (34). When combined, these modalities promote both peripheral adaptations, such as increased capillarization and mitochondrial density in working muscles, and central adaptations, including enhanced cardiac output and oxygen delivery efficiency (35, 36). This dual mechanism results in a comprehensive improvement in aerobic capacity. On the other hand, the functional training group also experienced substantial increases in VO2 max, though to a lesser extent than the combined group, as functional exercises inherently involve high levels of cardiovascular demand and dynamic movement patterns, which are well-suited for stimulating aerobic adaptations (37). One research demonstrated that combined training significantly enhances VO₂ max compared to traditional or functional training modalities. This superior improvement can be attributed to the synergistic physiological adaptations elicited by concurrent aerobic and resistance stimuli. Aerobic exercise augments cardiovascular efficiency and mitochondrial density, while resistance training enhances muscular strength and oxygen utilization. The integrative effect promotes greater cardiorespiratory endurance, evidenced by elevated VO₂ max values, reflecting improved maximal oxygen uptake and systemic metabolic efficiency in overweight individuals (38). However, the absence of the strength component may limit the development of maximal oxygen utilization potential. Conversely, the traditional training group exhibited minimal improvements in VO2 max, as its primary focus on resistance-based exercises does not significantly challenge the cardiovascular system (34, 39). Although some peripheral adaptations, such as improved oxygen extraction at the muscle level, may occur, the absence of sustained aerobic stimuli limits significant gains in cardiorespiratory fitness (40). Finally, the control group showed no significant changes, reinforcing the necessity of structured and targeted physical activity to induce physiological adaptations.

The findings of this study indicate that the combination of traditional and functional training elicited the most substantial improvements in strength compared to the other groups, likely due to the synergistic effects of these complementary training modalities. Traditional strength training typically involves structured, high-resistance exercises targeting specific muscle groups, thereby promoting hypertrophy and neuromuscular adaptations (41). On the other hand, functional training emphasizes movements that replicate real-life activities or sport-specific patterns, improving intermuscular coordination, balance, and stability (42). When combined, these approaches create a multidimensional stimulus that optimizes both muscular and neural adaptations, enhancing overall strength gains more effectively than either method alone (43). The functional training group also demonstrated significant strength improvements, albeit less pronounced than the combined group, possibly due to its focus on dynamic and compound movements, which prioritize motor control and joint stability over maximal force production (44, 45). In contrast, the traditional training group exhibited only minimal strength gains, as its isolated and repetitive nature may have limited the scope of neuromuscular adaptations, particularly in areas such as proprioception and dynamic stability (46). Meanwhile, the control group showed no significant differences, underscoring the necessity of structured physical training to induce measurable physiological changes.

The findings of this study underscore the superior efficacy of combined traditional and functional training in reducing body fat percentage, compared to functional training alone, traditional training alone, and no intervention (control group). The marked decrease in body fat percentage in the combined training group attributed to the synergistic effect of integrating the benefits of both training modalities (47). Traditional strength training, characterized by its emphasis on progressive overload and high-intensity resistance exercises, is known to enhance basal metabolic rate and promote lean muscle mass, which in turn elevates energy expenditure even at rest (48). Functional training, on the other hand, incorporates dynamic, multi-joint movements that replicate real-life activities and typically engage multiple muscle groups simultaneously, leading to an increased caloric burn during sessions and improved neuromuscular efficiency (49). The combination of these two methods likely maximizes fat oxidation by blending the metabolic benefits of traditional training with the functional benefits of dynamic, high-intensity movements. The functional training group exhibited a moderate reduction in body fat percentage, which is consistent with previous research highlighting its effectiveness in increasing energy expenditure and improving body composition (50). A study by Wang et al. (19) demonstrated synergistic approach likely potentiates lipolytic processes through complementary physiological pathways: aerobic training enhances mitochondrial density and fat oxidation capacity, while resistance elements stimulate muscle protein synthesis and elevate post-exercise metabolic rate. The resultant increase in fat-free mass creates a more favorable metabolic environment for adipose tissue reduction (19). The absence of the progressive overload component characteristic of traditional training limit its potential to sustain prolonged post-exercise metabolic elevation (51). In contrast, the minimal changes observed in the traditional training group alone could be due to the lower energy expenditure during resistance exercises as compared to the functional training group, coupled with the potential adaptation of the body to repetitive, isolated movements over time, which blunt the fat-loss response (47). The lack of significant improvement in the control group emphasizes the critical role of physical activity in body composition management, as sedentary behaviors are often associated with reduced energy expenditure and a propensity for fat accumulation (52). This study highlights the importance of a multi-dimensional approach to training for optimal body composition outcomes and provides a compelling argument for incorporating functional elements into traditional training programs to maximize fat loss efficiency.

The remarkable improvement in the combined training group indicated the synergistic integration of traditional strength-based protocols and functional training exercises. Traditional training, often emphasizing hypertrophy, maximal strength, and neuromuscular adaptation, provides a robust foundation for muscle force production and structural resilience (53). Conversely, functional training focuses on enhancing sport-specific movements, neuromuscular coordination, and dynamic stability, which are crucial for optimizing sprint performance. When these modalities are combined, the resultant training stimulus enhances both the foundational attributes (e.g., muscular strength and power) and task-specific biomechanical efficiency, allowing for a more comprehensive improvement in sprinting mechanics (54). In contrast, the group undergoing functional training alone also demonstrated notable gains, likely due to its emphasis on dynamic multi-joint exercises that mimic sprinting movements, enhancing neuromuscular coordination and elastic strength (55). A systematic review demonstrated that combined training protocols yield superior improvements in coordination compared to isolated functional or traditional approaches. This synergistic effect likely stems from the neurophysiological benefits of varied stimuli, which enhance proprioceptive feedback mechanisms and neural pathway development. The integration of complementary training modalities appears to optimize both intramuscular and intermuscular coordination through enhanced sensorimotor integration (56). However, the absence of substantial overload from traditional strength exercises may have limited their capacity to generate maximal propulsive forces, explaining the relatively smaller improvement compared to the combined training group (57). Meanwhile, the traditional training group displayed minimal improvements, which could be due to its limited focus on dynamic movement patterns and sport-specific agility components. Sprinting is not solely dependent on maximal strength but also requires rapid force production, limb coordination, and efficient energy transfer, areas where traditional training alone falls short (53). The lack of significant improvement in the control group underscores the necessity of targeted interventions, as sprinting ability is not significantly enhanced without a structured training regimen (58). The exercises mimic sport-specific demands, enabling athletes to develop superior reactive capabilities, dynamic stability, and quick changes of direction, all of which are critical components of agility. In contrast, the combined training group, while showing notable improvements in sprinting ability, demonstrated less pronounced gains in agility (55). This is due to the inclusion of traditional strength training exercises in the combined protocol, which prioritize linear force production over the complex motor patterns required for agility (59). The sprinting improvements in the combined group suggest that integrating strength and functional training can enhance explosive power and acceleration, likely due to the synergistic activation of fast-twitch muscle fibers and improved rate of force development (54). However, the relatively lower focus on agility-specific drills in this protocol likely limited the group’s potential for agility enhancement. Meanwhile, the traditional training group and the control group did not exhibit significant improvements, which underscores the specificity principle of training in sports science (60). Traditional strength training, predominantly involving linear and isotonic movements, fails to replicate the dynamic and multidimensional demands of sports, thereby limiting its transferability to agility performance. Similarly, the lack of structured training stimulus in the control group naturally precluded any meaningful physiological or neuromuscular adaptations.

Traditional strength training primarily focuses on augmenting muscle strength, power, and endurance through isolated, controlled movements that build the foundational physical capacities required for coordinated actions. Functional training emphasizes movement patterns that replicate real-life activities and sport-specific actions, integrating multiple muscle groups and joints in dynamic and multidirectional contexts (61). This dual approach in the combined training group likely stimulated both the central and peripheral nervous systems, fostering improved intermuscular and intramuscular coordination, motor control, and movement economy (62). Functional training alone demonstrated moderate improvements in coordination due to its emphasis on dynamic stabilization, agility, and proprioception, but it lacked the robust strength foundation provided by traditional methods, which may limit its overall impact (63). Meanwhile, traditional training alone showed minimal gains in coordination, as its isolated exercises do not sufficiently challenge the proprioceptive and neuromuscular adaptations required for enhanced movement integration. The control group, which did not undergo any specific training intervention, exhibited no significant improvement, underscoring the necessity of targeted training stimuli for physiological and neurological adaptations (64, 65). Collectively, these findings highlight the superior efficacy of combining traditional and functional training methods to optimize coordination, as the complementary adaptations generated by these modalities address both foundational strength and functional movement demands, which are crucial for athletic performance and injury prevention in sports contexts. Despite the comprehensive design and significant findings, this study is not without limitations. Firstly, the duration of the intervention was limited to 8 weeks, which may not capture long-term adaptations or sustainability of gains. Secondly, dietary habits and recovery protocols were not strictly controlled, which could have influenced physiological responses. Thirdly, the sample was restricted to adolescent male athletes from a specific region, limiting the generalizability to other age groups, female athletes, or different populations. Additionally, psychological variables such as motivation and fatigue were not assessed, which could have influenced training performance. Future research should aim to address these limitations through longer interventions, diverse populations, and holistic monitoring of training conditions.

## 5 Conclusion

This study aimed to evaluate and compare the effects of functional training (FT), traditional training (TT), and their combination (CT) on sprint performance and related physical fitness parameters among adolescent sprinters. The findings clearly demonstrate that combined training (CT) is the most effective approach, producing superior improvements across all measured variables, VO₂ max, muscular strength, body composition, sprinting ability, agility, and coordination, when compared to FT, TT, or no intervention. While FT enhanced agility and coordination, and TT improved strength moderately, only the integrated CT approach produced comprehensive gains in both physiological and skill-related performance. These results affirm that combining FT and TT in a structured, periodized model optimally addresses the multifaceted demands of sprint performance in adolescent athletes.

## Data Availability

The original contributions presented in the study are included in the article/supplementary material, further inquiries can be directed to the corresponding author.
